# HexSE: Simulating evolution in overlapping reading frames

**DOI:** 10.1093/ve/vead009

**Published:** 2023-02-23

**Authors:** Laura Muñoz-Baena, Kaitlyn E Wade, Art F Y Poon

**Affiliations:** Department of Microbiology and Immunology, Western University, Dental Sciences Building 3014, London N6A 3K7, Canada; Department of Pathology and Laboratory Medicine, Western University, Dental Sciences Building 4044, London N6A 5C1, Canada; Department of Microbiology and Immunology, Western University, Dental Sciences Building 3014, London N6A 3K7, Canada; Department of Pathology and Laboratory Medicine, Western University, Dental Sciences Building 4044, London N6A 5C1, Canada

## Abstract

Gene overlap occurs when two or more genes are encoded by the same nucleotides. This phenomenon is found in all taxonomic domains, but is particularly common in viruses, where it may provide a mechanism to increase the information content of compact genomes. The presence of overlapping reading frames (OvRFs) can skew estimates of selection based on the rates of non-synonymous and synonymous substitutions, since a substitution that is synonymous in one reading frame may be non-synonymous in another and vice versa. To understand the impact of OvRFs on molecular evolution, we implemented a versatile simulation model of nucleotide sequence evolution along a phylogeny with any distribution of open reading frames in linear or circular genomes. We use a custom data structure to track the substitution rates at every nucleotide site, which is determined by the stationary nucleotide frequencies, transition bias and the distribution of selection biases (dN/dS) in the respective reading frames. Our simulation model is implemented in the Python scripting language. All source code is released under the GNU General Public License version 3 and are available at https://github.com/PoonLab/HexSE.

## Introduction

Overlapping reading frames (OvRFs) are portions of the genome where the same nucleotide sequence encodes more than one protein. They have been documented across all taxonomic domains including bacteria, vertebrates, and fungi (Gerads and Ernst 1998; Chung et al. 2007; Pallejà, Harrington and Bork 2008; Ribrioux et al. 2008). OvRFs are particularly abundant in virus genomes and examples can be found in all Baltimore classes (Muñoz-Baena and Poon 2022). In viruses, OvRFs may provide a mechanism to store more information in smaller genomes, to make purifying selection more efficient at removing deleterious mutations, or to facilitate the *de novo* creation of genes (Krakauer and Plotkin 2002; Belshaw et al. 2008; Chirico, Vianelli and Belshaw 2010; Sabath, Wagner and Karlin 2012; Willis and Masel 2018). OvRFs can be classified into six different frameshifts that can be annotated as +2, +1, +0, −0, −1, −2 (Lèbre and Gascuel 2017), although other labeling schemes exist. Here, the sign indicates whether the overlap occurs between open reading frames (ORFs) on the same (+) or the opposite (−) strands, and the integer indicates how many nucleotides they are shifted relative to one another. Frameshifts influence the effect of selection in OvRFs. For example, a +0 frameshift can amplify selection on codons that become expressed in multiple contexts without changing the number of nucleotides under selection. However, measuring selection within OvRFs is a difficult problem because the effect of a nucleotide substitution (i.e., whether synonymous or non-synonymous) depends on multiple codon contexts (Pedersen and Jensen 2001).

Computer simulations of biological processes are widely used as a tool to characterize biological processes that are otherwise too complex to represent as a mathematical model for analysis (Arenas 2012). There is a great diversity of programs that simulate molecular evolution along a phylogeny (Rambaut and Grass 1997), which are designed to model different aspects of evolution including recombination (Cartwright 2005), insertions and deletions (Strope, Scott and Moriyama 2007), variable selection intensities across the sequence (Hall 2008), and different substitution biases (Spielman and Wilke 2015). However, we have not found a publicly available program that can simulate evolution in linear or circularized genomes with an arbitrary distribution of overlapping and non-overlapping genes and variable codon-specific selection pressures, yielding a multiple sequence alignment for a given tree. A standard simplifying assumption in molecular evolution is that nucleotides or codon substitutions are independent and identically distributed outcomes of a continuous-time Markov model. However, a codon within an OvRF is no longer independently evolving, since a nucleotide substitution can change the selective context for subsequent changes at nucleotides of adjacent codons.

**Figure 1.**
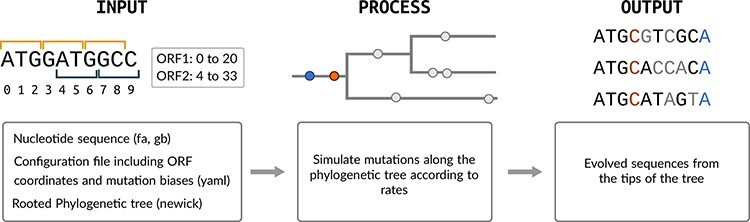
Pipeline overview. We use an exact stochastic simulation algorithm to simulate the accumulation of nucleotide substitutions in the selective context of one or more ORFs, some of which may overlap. The simulation is initialized with an input sequence at the root (left panel), and traverses branches of the phylogenetic tree (center panel) in order to generate a nucleotide alignment at the tips (right panel).

Here, we describe a simulation method (HexSE, Fig. 1) implemented in Python that simulates molecular evolution along an input tree where the sequence may contain any number of OvRFs. We employ a memory-efficient data structure to track the rates of substitution events at every individual nucleotide of an evolving sequence.

## Implementation

### Input specification

HexSE takes three input files. First, it requires a FASTA- or GenBank-formatted file containing a nucleotide sequence (minimum 9 nt) to seed the simulation at the root of the tree. Next, the user must provide a configuration file in the form of a YAML-formatted file specifying the location of each ORF, including the parameters of the distribution that will be used to sample the frequency of mutations for each gene. Lastly, HexSE requires a file containing the Newick serialization of a phylogenetic tree. The tree must be rooted, i.e., contain a root node to assign the input sequence. In addition, the tree must contain branch lengths. These lengths can be rescaled by the user by adjusting the global mutation rate parameter. For example, the program will simulate 0.01 substitution events per site on average along a branch of length 1 if the global mutation rate is set to 0.01 (notwithstanding other rate adjustments such as selection).

#### Configuration file

Additional parameters that may be specified in the YAML-formatted control file include whether the genome is circular or not (default: false); a global transition/transversion ratio *κ* (default: 0.3); the parameters and number of rate categories for discretized gamma or lognormal distributions, which are used to model variation in selection (*ω*) and mutation rates (*µ*) for each reading frame; and the stationary nucleotide frequencies *π*. By default, *π* is set to the empirical frequencies in the root sequence.

### Algorithm

To simulate sequence evolution, we use a standard Gillespie (1976) algorithm for the exact stochastic simulation of discrete events (nucleotide substitutions). The model is initialized by assigning the input sequence to the root of the tree and calculating the rates of every possible substitution at position *i* to nucleotide *j*:

$$ \lambda_{ij} = \begin{cases} \mu_i\pi_j & \text{for a completely synonymous or non-coding}\\ & \text{transversion,}\\ \mu_i\kappa\pi_{j} & \text{for a completely synonymous or non-coding}\\ & \text{transition,}\\ \mu_i\omega\pi_j & \text{for a non-synonymous transversion,} \\ \mu_i\omega\kappa\pi_j & \text{for a non-synonymous transition,} \\ \end{cases} $$

where *µ*_*i*_ is the baseline mutation rate. The effect of selection is tracked by a vector **w** corresponding to the reading frames $\{-2,-1,-0,+0,+1,+2\}$, such that $\|\mathbf{w}\|=6$. The *i*-th element of **w** is set to $w_i=1$ if the substitution is synonymous in that reading frame. If $w_i\gt1$, non-synonymous mutations accumulate more rapidly on average than synonymous mutations, suggesting that the codon is under positive diversifying selection. Conversely, if $w_i\lt1$, non-synonymous mutations accumulate at a lower rate, indicating that the site is under negative (purifying) selection. In the absence of empirical information on how selective effects in overlapping reading frames combine, we assume that these effects are multiplicative. Therefore, the total selective effect on a substitution in HexSE is $\omega=\prod \mathbf{w}$. If a substitution is in a non-coding region or synonymous in all six reading frames, then *ω* = 1, i.e., the substitution evolves neutrally.

Next, we draw an exponentially distributed waiting time to the next event, $t\sim \exp(-\Lambda)$, where $\Lambda = \sum_{ij} \lambda_{ij}$. If *t* exceeds the length of the current branch, no event occurs and the current sequence is propagated to the next node. Otherwise, a substitution event is drawn with a probability proportional to the associated rate, $\lambda_{ij}/\Lambda$, and the sequence is updated by that event. Ultimately, the substitution rates are re-calculated for the updated sequence. This process continues by level-order traversal of the phylogenetic tree until every terminal node (tip) of the tree carries an evolved sequence.

### Simulating a mutation

**Figure 2.**
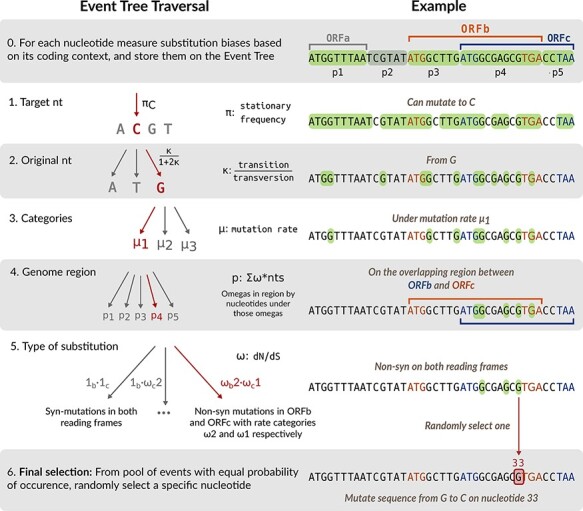
**Traversal of an event probability tree to select mutations.** An event probability tree is a data structure that we used to store nucleotide substitutions with the same probability. Each level of the event tree corresponds to an evolutionary parameter, such as the transition/transversion rate bias. Each branch represents a discrete value associated with the parameter represented by that level. Traversing the event tree from the root (left panel) selects progressively smaller subsets of mutations, as demonstrated in the right panel.

Computing and storing the rates of every possible substitution is time-consuming and selecting the next substitution event by drawing a uniform random number would require a linear search of the cumulative probabilities. Since we use a discretized probability distributions for *ω* and *µ*, these rates can only assume a finite number of values. Therefore, substitution events can be rapidly selected by traversing a hierarchical data structure that we call an *event probability tree*. All possible substitution events are stored at the tips of the event tree (Fig. 2). Starting at the root of the event tree, we select the target nucleotide *j* with probability *π*_*j*_, moving down to the respective node at the next level. Next, we select the starting nucleotide *k* with probability $1/(1+2\kappa)$ if $k\rightarrow j$ is a transition and $\kappa/(1+2\kappa)$ otherwise. We select the mutational rate category given the global rate distribution for *µ*, followed by selecting a particular region of the genome. When initializing the run parameters, we divide the sequence into categories determined by the distribution of ORFs. For example, we divide the nucleotide sequence depicted in Fig. 2 into five regions: nucleotides occurring within ORF *a* only ($p1$); ORF *b* only ($p3$); the overlapping region between ORF *b* and ORF *c* ($p4$); ORF *c* only ($p5$), and nucleotides that are not in any ORF ($p2$).

The probability of selecting a region is proportional to the number of potential substitution events weighted by the net effect of selection, which is determined by the rate categories (distributions) of *ω*s associated with every ORF in the region. The weighted sum is calculated as $\sum_k \omega_{k} N_k$, where *N*_*k*_ is the number of nucleotide sites associated with the *k*-th combination of selection biases *ω*_*k*_, and the sum is computed over all such combination in the region. For example, a substitution at position *i* that is non-synonymous in ORFs *a* and *b* with reading frames −1 and +0 can have a vector $\textbf{w}=\{1, \omega_3^a, 1, \omega_2^b, 1, 1\}$, where $\omega_l^g$ is drawn from a discretized distribution associated with ORF (gene) *g*. The value of *ω*_*k*_ at position *i* results from multiplying the values assigned to $\omega_3^a$ and $\omega_2^b$.

Finally, we choose one of the *ω* combinations in the region, with a probability determined by the combination of selection rate biases. If a nucleotide is involved in a ‘start’ or ‘stop’ codon, then we do not include it in the event tree to prevent mutations from disrupting ORFs.

Traversing the event probability tree from the root to a tip resolves the shared characteristics for a subset of substitution events. In other words, the tip stores references to the nucleotide substitution events that have the same probability of occurring. From this subset we can simply select an event uniformly at random and update the evolving sequence accordingly. Since the composition of event subsets at the tips of the tree are updated with the sequence, we perform a deep copy of the event probability tree at every internal node of the phylogeny.

### Simulating evolution in the HBV genome

To demonstrate the usage of HexSE, we simulated evolution in the hepatitis B virus (HBV) genome, where around 30 per cent of nucleotides within ORFs are involved in overlaps. Our inputs for the simulation were the HBV reference genome (accession NC_003977.2) in GenBank format including coding sequence annotations for genes S, P, C, and X; a random phylogenetic tree with 100 tips, using the implementation of the Kuhner and Felsenstein (1994) algorithm in T-REX (Boc, Diallo and Makarenkov 2012); and the substitution model parameters. These parameters included a global mutation rate of 0.05, two rate categories for *µ* drawn from a lognormal distribution with two classes and shape 1.0 (resulting in rates 0.026 and 1.39 substitutions per nucleotide per unit time), a transition–transversion rate ratio of 0.3, and varying distribution parameters for *ω* in the four ORFs. A second run was performed under the same parameters, but excluding all ORFs except gene C (nucleotide coordinates 1,816 to 2,454).

Levels of genetic variation in simulation outputs varied substantially among codon sites, depending on their protein-coding context. We quantified these changes in genetic diversity using the Shannon entropy, $H=-\sum_{i=1}^{M} P_i\,\log_2\,P_i$, where *M* is the number of residues and *P*_*i*_ is the relative frequency of the *i*-th residue in the alignment column. This statistic provides a generic measure of diversity that applies to any combination of reading frames. We calculated the mean nucleotide entropy for sliding windows along the alignment with a width of 20 nucleotides and a step size of 1 nucleotide. For nucleotide residues, this statistic varies from zero, for a completely conserved site, to a maximum of *H* = 2.

**Figure 3.**
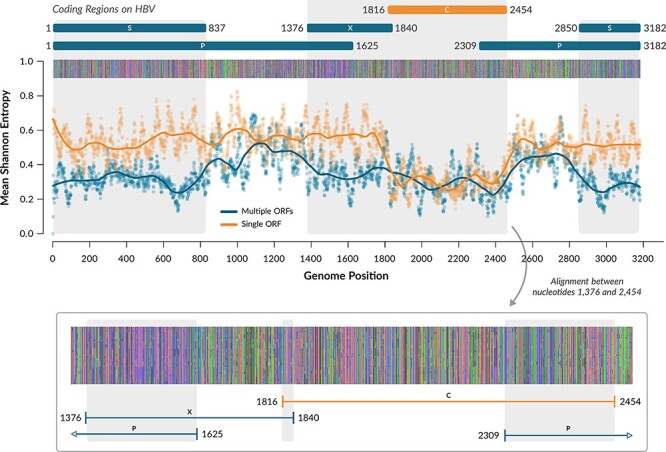
**Simulating Evolution on HBV.** Using HexSE, we simulated evolution in the HBV reference genome, with all four ORFs (blue) or only one ORF (orange). We calculated the nucleotide entropy per site for the resulting alignments to account for the number of mutations accumulated on each position over time. The region encoding Gene C has similar entropy values for both simulations. However, in the simulation with all ORFs, the mean entropy falls from a mean of 0.306 to 0.222 in the overlapping region (nucleotides 2,309 to 2,454). There, mutations rarely accumulate, even in comparison with adjacent regions, which are also protein-coding regions. In general, overlapping sites tend to accumulate less mutations than non-overlapping sites of the genome. This pattern is the most evident in the transition from the non-overlapping regions of Gene P (nucleotides 837 to 1,376 and 2,454 to 2,850) to the region overlapping gene S (nucleotides 2,850 to 837).

A comparison of mean nucleotide entropy profiles between two simulation outputs on HBV sequences with one and four ORFs, respectively, is shown in Fig. 3. In the simulation results where we included only one gene, we detected higher entropy in the non-coding regions (mean 0.539, interquartile range (IQR) 0.475−0.598), which is consistent with the net effect of purifying selection in the coding region of Gene C (mean 0.302, IQR 0.254−0.342). We observe similar mutation patterns in the non-overlapping region of Gene C, on the alignment produced when all four ORFs are included. This region, comprised between nucleotides 1,840 and 2,309, has a mean of 0.306 (IQR 0.247−0.366) and reaches a minimum of 0.104. As expected, we noticed a significant difference in the entropy levels between overlapping and non-overlapping regions (average 0.304 vs. 0.397; Wilcoxon rank sum test, $P\lt10^{-15}$). These shifts in mean entropy are also confounded by variation among ORFs in the intensity of purifying selection, as well as stochastic variation. These patterns are consistent with the cumulative effect of purifying selection in one or more ORFs on limiting diversification at the level of nucleotide sequences.

The average time that HexSE requires to simulate evolution on HBV under our example parameters was 52.74 s (IQR: 48.36−58.01 s, *n* = 20) using four cores on an AMD Ryzen 5 3400 g processor, with Python 3.6.9 and Ubuntu 18.04.6. In contrast, the same simulation took about 310.3 s (IQR 281.3–308.1) to complete on a tree with 500 tips. In general, running times scaled linearly with respect to the number of tips on the phylogenetic tree (Supplementary Fig. S1).

### Accounting for variation in selection intensities

To assess HexSE’s ability to generate realistic outputs, we compared selection pressures on HBV Gene C between two independent HexSE runs and a curated multiple sequence alignment of 170 complete genomes of HBV Genotype F (Bell, Yousif and Kramvis 2016). We used the alignment of actual HBV sequences to reconstruct a maximum-likelihood phylogenetic tree using FastTree (version 2.1.10) (Price, Dehal and Arkin 2010). Both HexSE runs were initialized with the resulting tree and the consensus sequence of the alignment. We used the accessory script gb_to_yaml.py to configure the run specification files based on the annotation of the HBV genome sequence KY382417.1. Thus, both configuration files included the information required to simulate selection in the four ORFs that encode Proteins S, P, X, and C in HBV. The global mutation rate was set to 6, and the transition–transversion rate ratio (*κ*) to 0.3, for both runs.

To parameterize *ω* in HexSE, users can modify the shape (*α*) and scale (*β*) parameters of the gamma distribution, considering that the mean of this distribution is $\alpha/\beta$. For the first run, for example, we set these parameters to *α* = 2 and *β* = 0.5, respectively, and set the number of categories to four in the ORF that encodes Gene C. The resulting mean is 1 and the values associated with the four equiprobable categories are 0.293, 0.655, 1.069, and 1.981. Hence, only one-quarter of sites in the ORF would experience positive selection. We followed the same approach for Genes X, P, and S.

**Figure 4.**
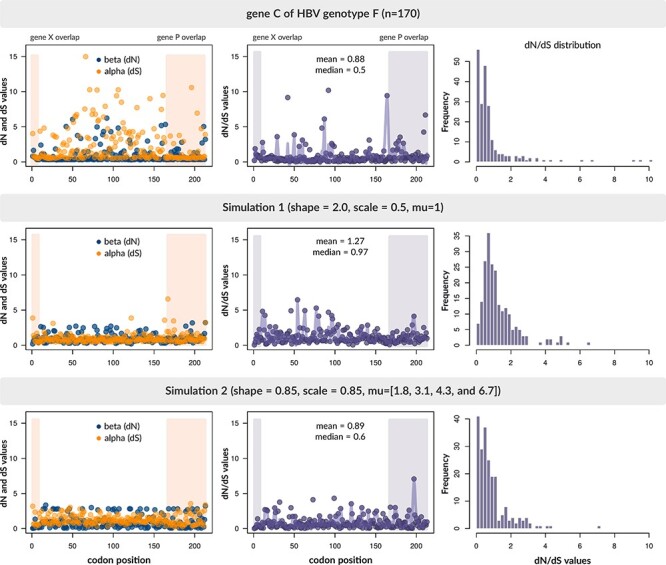
Comparison of selection pressures on between Gene C of HBV Genotype F and two simulated alignments. We used the FUBAR method to estimate selection intensities in the genome region comprised between nucleotides $1,813$ and $2,452$ of a real alignment of HBV Genotype F, and two independent HexSE simulations. For the first simulation, we selected values for the shape and scale parameters based on the uninformed assumption that the majority of codon sites were under neutral or purifying selection. In contrast, we parameterized the second run based on the empirical estimates of dN/dS calculated on the codon sequences of Genes X, P, and C.

In contrast, the second run was parameterized using Fast Unconstrained Bayesian Approximation (FUBAR) (Murrell et al. 2013) to estimate dN and dS values for the real alignment. In HBV Genotype F, the ORF that encodes gene C (reference coordinates 1,813 to 2,452) overlaps in 25 nucleotides with Gene X (coordinates 1,373 to 1838) and in 146 nucleotides with Gene P (coordinates 2,306 to 3,215; 0 to 1,623). We fitted a gamma distribution to the selection patterns in the codon sequences of Genes X, P, and C and run the second simulation based on these empirical estimates. Additionally, we introduced variation to the mutation intensities by drawing mutation rates (*µ*) from a gamma distribution with *α* = 2 and *β* = 1 (resulting in four categories of 1.8, 3.1, 4.3, and 6.7). As expected, the distribution of dN/dS values for the second simulation run was more similar to the actual data ($\hat{\alpha}=0.85$ and $\hat{\beta}=0.85$; Fig. 4). Because our simulations constrained dS to 1, however, we also observed a lack of variation in dS values compared to real data that were only partially compensated by adding variation in mutation rates.

## Supplementary Material

vead009_Supp
