# Supplementary figures and images for: HexSE: Simulating evolution in overlapping reading frames
Source: Virus Evol. 2023 Feb 23;9(1):vead009. doi: 10.1093/ve/vead009 (PMC9949996; doi:10.1093/ve/vead009)

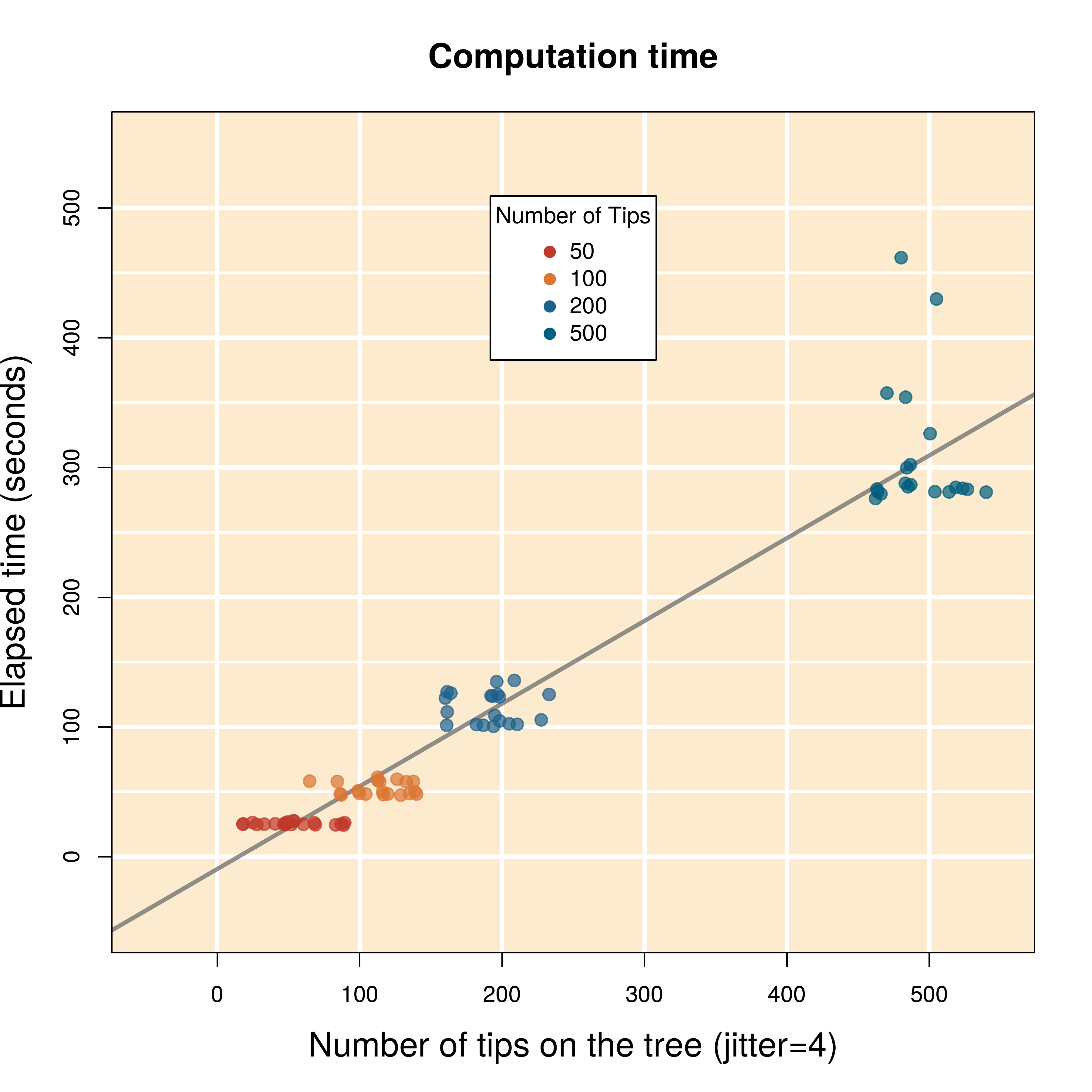

Supplement: vead009_Supp [file vead009_supp.zip › FigureS1.tiff]
